# Visually and olfactorily enhanced attractive devices for thrips management

**DOI:** 10.1111/eea.12969

**Published:** 2020-09-27

**Authors:** Robert W. H. M. van Tol, Melanie M. Davidson, Ruth C. Butler, David A. J. Teulon, Willem Jan de Kogel

**Affiliations:** ^1^ Wageningen University and Research PO Box 69 Wageningen 6700 AB The Netherlands; ^2^ New Zealand Institute for Plant and Food Research Limited Private Bag 4704 Christchurch New Zealand

**Keywords:** *Frankliniella occidentalis*, *Thrips tabaci*, *Thrips obscuratus*, colour, methyl isonicotinate, semiochemical, Thysanoptera, Thripidae, monitoring, olfaction, lure‐and‐infect, pest management strategy, sticky plate trap, auto‐dissemination

## Abstract

‘Lure‐and‐infect’ is an insect pest management strategy with high potential but so far there are few examples of its application. Using traps as surrogates for auto‐dissemination devices, we tested the attractiveness to naturally occurring thrips (Thysanoptera: Thripidae) of three trap types differing in colour and structure, with and without the thrips lure methyl isonicotinate (MI), and sticky plate traps as a control. The aim was to find more effective traps that could be further developed into devices for auto‐dissemination and lure‐and‐infect of thrips. The number of thrips captured varied substantially with trap type and the presence of the MI lure. We found a high visual response to a sticky ‘white ruffle’ trap (i.e., a 30‐cm‐long cylindrical outline of folded fabric), compared to a commonly used blue sticky plate trap (Bug‐scan) as the control. This effect was seen both in a greenhouse with roses (*Rosa* spp.), where we encountered western flower thrips, *Frankliniella occidentalis* (Pergande), and in a grass field, where we encountered onion thrips, *Thrips tabaci* Lindeman, and New Zealand flower thrips, *Thrips obscuratus* (Crawford). In the absence of MI, the white ruffle trap caught 7–22× more thrips than the control Bug‐scan trap. A similarly designed blue ruffle trap and a modified Lynfield trap caught lower thrips numbers than the white ruffle and the control Bug‐scan traps. Presence of MI substantially increased the captures of *T. tabaci* in all three trap types in the field (2.5–18×). In the greenhouse, without MI the white ruffle trap caught 3.5–14× more thrips than the Bug‐scan, blue ruffle, or modified Lynfield traps. Presence of MI increased the captures of *F. occidentalis* males and females in the Lynfield and blue ruffle traps (1.4–2.8×), but not in the white ruffle trap in the greenhouse (ca. 1.1×). The importance of visual and olfactory factors for the design of effective auto‐dissemination and lure‐and‐infect strategies for thrips management is discussed.

## Graphical abstract

There are few examples of ‘lure‐and‐infect’ as a pest management strategy. We compared the attractiveness to naturally occurring thrips of three trap types differing in colour and structure, with and without the thrips lure methyl isonicotinate. A white ruffle trap attracted more thrips than a blue ruffle trap, a modified Lynfield device, and control sticky plates. The lure increased thrips captures in all trap types. Visually and semiochemically enhanced traps may improve the lure‐and‐infect strategy for thrips.

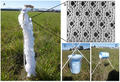

## Introduction

‘Lure‐and‐kill’ and ‘lure‐and‐infect’ are two insect pest management strategies that combine an insect lure with a killing agent that works over a short time period (e.g., an insecticide), or a lethal disease that can be transmitted to the wider population [e.g., an entomopathogenic fungus (EPF)] respectively (Cork, 2016; Yousef et al., 2018). The objective of lure‐and‐infect is not the immediate death of the infected insect, but its return to the population to infect conspecifics with the pathogen (i.e., auto‐dissemination or auto‐inoculation) (Vega et al., 2007). For both lure‐and‐kill and lure‐and‐infect, an effective lure or attractive device is necessary (i.e., an auto‐dissemination device), where the insects can come into contact with either the insecticide or the pathogen (e.g., fungal spores).

Gregg et al. (2018) describe several successful lure‐and‐kill options with insecticidal compounds. However, lure‐and‐infect is rarely mentioned as a successful pest control strategy, for a variety of possible reasons. The method requires contact between EPF spores and the insect, which may be hampered by insect behaviour (e.g., limited walking on surface with spores) or surface limitations (e.g., a flat surface only allows for infection of insect legs). Also, transfer of spores to conspecifics is critical. The auto‐dissemination device should have olfactory (e.g., semiochemical) and visual (e.g., colour) cues that are effective enough to attract insects, and the device should promote landing and/or walking behaviour. In the present study we focus on insect attraction, and aim to increase attractiveness of the visual and olfactory cues of auto‐dissemination devices to several species of thrips (Thysanoptera: Thripidae).

Insect pathogens such as the fungi *Metarhizium anisopliae* (Metschn.) Sorokīn and *Beauveria bassiana* (Bals.‐Criv.) Vuill. can be effectively used in auto‐dissemination devices baited with insect attractants or lures (Migiro et al., 2010; Navarro‐Llopis et al., 2015). There is only one published description of an auto‐dissemination device for a lure‐and‐infect strategy of thrips, a modified Lynfield trap, in which an EPF is used as the ‘infect’ component. Its effectiveness against trips has been reported in several publications (Dimbi et al., 2003; Migiro et al., 2010; Niassy et al., 2012; Mfuti et al., 2016). A (visually) more attractive device may attract and arrest movement of more thrips, which may lead to more contact between thrips and EPF spores, which may in turn lead to more successful auto‐dissemination or infection and transfer of spores to conspecifics (Quesada‐Moraga et al., 2008; Yousef et al., 2018).

The colour preference of a key pest species, western flower thrips [*Frankliniella occidentalis* (Pergande)], has long been debated. In comparative field and greenhouse studies, mainly three colours – blue, yellow, and white – are effective for trapping this species (Johansen et al., 2018). The trap colour that catches the most thrips varies between locations and crops, and the causes of these underlying differences are not well understood.

A few studies have examined ‘enhanced’ colours (fluorescence) for trapping thrips. Fluorescence is the transition of ultraviolet (UV) light into other colours, which may increase the brightness of traps (Marshall & Johnsen, 2017). Jenser et al. (2010) found that fluorescent yellow traps increased the captures of *Drepanothrips reuteri* Uzel in grape (*Vitis vinifera* L.) compared to several non‐fluorescent colours, but Devi & Roy (2017) found that fluorescent green was not as effective as (non‐fluorescent) blue for onion thrips (*Thrips tabaci* Lindeman) in onion (*Allium cepa* L.). Röth et al. (2016) found high attraction of western flower thrips in winter to a yellow fluorescent trap, but not in late winter/spring, when blue appeared as attractive.

Ultraviolet reflection from white traps may also affect thrips captures. White traps that reflect more UV light catch fewer western flower thrips and onion thrips than white traps that reflect less UV light (Kirk, 1984; Hoddle et al., 2002), although other studies report the opposite (Matteson & Terry, 1992; Makabe et al., 2014). Little is known about the role of UV reflection in relation to other colours. Matteson & Terry (1992) found that UV reflection values below 35% had no effect on thrips captures for blue and yellow traps, and they found no correlation between UV brightness and thrips captures. Most of the commercial coloured traps we have tested over the years absorb most of the UV light, and do not reflect it (RWHM van Tol, unpubl.).

Kirk et al. (2021) summarize numerous compounds that increase thrips captures on traps. A more universal lure of several important pest thrips, such as the western flower thrips and the onion thrips, is methyl isonicotinate (MI) (Teulon et al., 2017; Kirk et al., 2021). This non‐pheromone compound not only increases captures of both male and female *F. occidentalis* and *T. tabaci*, but also several other pest thrips species (Teulon et al., 2017). Methyl isonicotinate is a putative semiochemical commonly called a kairomone (in a pest management context) but is probably more accurately described as a synomone (in an ecological context), as both the plant (pollination) and thrips (feeding and oviposition sites) can benefit from the interaction (Teulon et al., 2017). The compound has been shown to have potential in a lure‐and‐infect strategy with an EPF (Niassy et al., 2012). We therefore chose MI as olfactory lure in our research.

Surface contact in auto‐dissemination devices can be increased by using non‐woven and/or folded fabric, which may increase the contact of fungal spores with target insects (Baverstock et al., 2010). Folded fabric allows for a smaller device and is attractive for thrips because of their cryptic behaviour. In our study we compared four trap types. We developed a trap with folded fabric – known as WeevilGrip (Agri Gripping, Uithoorn, The Netherlands), effective for trapping root weevils (*Otiorhynchus* spp.) (Bruck et al., 2018; van Tol et al., 2020) – in two colour versions, blue and white. The third trap was a blue modified Lynfield trap (Niassy et al., 2012; Mfuti et al., 2016), and the fourth (control) trap was based on a blue Bug‐scan sticky plate trap (Biobest, Westerlo, Belgium). These traps were used as surrogates for auto‐dissemination devices and did not contain an actual pathogen.

We explored several aspects of auto‐dissemination devices to improve the efficacy of the first stage (i.e., insect approaching and landing) of lure‐and‐infect strategies for various naturally occurring pest thrips species in greenhouse and field experiments. We examined the effect of: (1) increase of the surface area of the devices, (2) ‘enhanced’ colour spectra, and (3) a semiochemical lure (MI) to increase thrips attraction. By applying an insect glue to the outer surfaces of the traps, we could estimate the number of insect landings, which is a precondition for uptake of spores. To compare the various devices, we calculated the trapping efficiency per unit of surface area.

## Materials and methods

### Auto‐dissemination devices and glue

**Figure 1:**
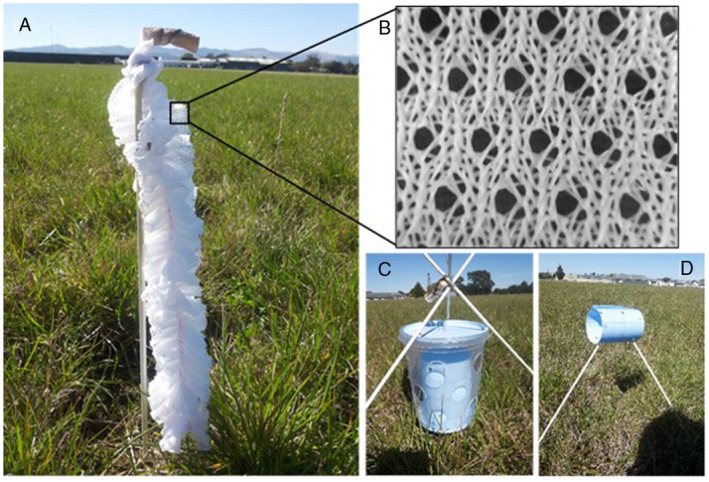
(A) White ruffle device with cotton role loaded with methyl isonicotinate on top, (B) details of the microstructure of the white ruffle device (the large holes in the fabric are 1 mm wide), (C) blue modified Lynfield device, and (D) blue Bug‐scan sticky plate trap rolled into a cylinder shape in the field. All devices were held in place at approximately 15 cm above the grass using fibreglass rods.

To study thrips attraction, the first stage of auto‐dissemination, we compared four auto‐dissemination trap types treated with insect glue. (1, 2) A ruffle device, WeevilGrip (30 cm long, 4 cm diameter), consisting of six folded layers of 100% polyester fabric (Micro Mesh #1280; Nick of Time Textiles, Allentown, PA, USA) (Figure 1A). The fabric had 1 mm holes, regularly and evenly distributed approximately 2 mm apart (Figure 1B). Insect glue (Stikem Special^;^ Seabright Laboratories, Emeryville, CA, USA) was applied to the entire surface of the ruffle device. The ruffle device was made in two colour versions, white (with optical brighteners) and blue (no addition of fluorescing compounds). (3) A modified Lynfield device (Dimbi et al., 2003; Migiro et al., 2010) (Figure 1C), consisting of a clear plastic beaker (10 cm high, 8.5 cm diameter at the top, 7 cm diameter at the bottom) perforated with 15 holes (2.5 cm diameter) evenly distributed on the wall of the container, with a lid on top. The outside of the lid and the bottom of the beaker were painted in a blue (Gamma zijdeglans, basic P, colour code GN 053‐10; https://www.kleurenwaaier.nl/kleur/gamma‐nieuw‐GN‐053‐10) similar to the Bug‐scan control traps (below). In our trap, a blue sticky Bug‐scan plate trap (25 cm long, 10 cm wide; Biobest), covered on the external side with Stikem Special insect glue (Seabright Laboratories) and rolled into a cylinder (10 cm high, 7 cm diameter), was placed inside the container. Thus, in order for a thrips to be trapped on the Bug‐scan trap within the container, it first needed to pass through one of the holes in the container wall. The original Lynfield trap consists of a clear plastic cylindrical container with four evenly spaced entry holes around the wall of the trap. Typically, a lure and an insecticide strip are placed inside to attract and kill target fruit flies (Cowley et al., 1990). And (4) as a control trap, a blue Bug‐scan sticky plate trap was rolled into a cylinder with the glue on the outside, similar to trap type 3; unlike trap type 3, this control trap had no plastic container and the cylinder was placed horizontally (vertical in trap 3; Figure 1D).

All devices in the field were held in place using 3‐mm‐diameter fibreglass rods (Figure 1). The top of each blue or white ruffle device was placed 35 cm above the surface, and the lower ends of the ruffles were placed at ca. 5 cm above the surface. The modified Lynfield traps were suspended with the lids at an approximate height of 15 cm, and the bottom just above the surface. The Bug‐scan trap rolled into a cylinder was held by two rods horizontally at an approximate height of 15 cm. For the treatments with MI, lures were attached as described below. The identical devices in the greenhouse were hanging on a cord just above the crop.

### Spectral reflectance of trap material

**Figure 2:**
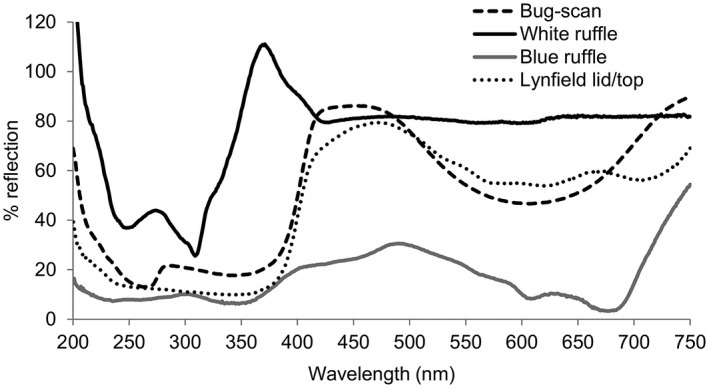
Spectral reflectance (%) – in the ultraviolet (UV) and visible light range – of material of the control blue Bug‐scan sticky plate trap, white ruffle, blue ruffle, and blue modified Lynfield devices. Insect glue was removed before determining the reflectance. For the modified Lynfield device, the reflectance of the painted lid of the bucket was measured.

The spectral reflectance of all materials used as colour cues was measured as previously described by Taylor et al. (2014), using a Thermo Evolution 220 UV‐visible spectrophotometer (Thermo Fisher Scientific, Waltham, MA, USA) (Figure 2). Samples were scanned using an ISA‐220 integrating sphere accessory in the sample compartment. Surfaces were scanned at wavelengths from 200 to 750 nm (UV and visible light up to infrared). Before scanning, all glue was removed from trap samples (4 cm long, 4 cm wide) using De‐Solv‐it (RCR International, Victoria, Australia), because the glue would have damaged the instruments. To avoid any background interference for the samples of ruffle fabric, four pieces from individual samples were stacked one on top of the other.

**Figure 3:**
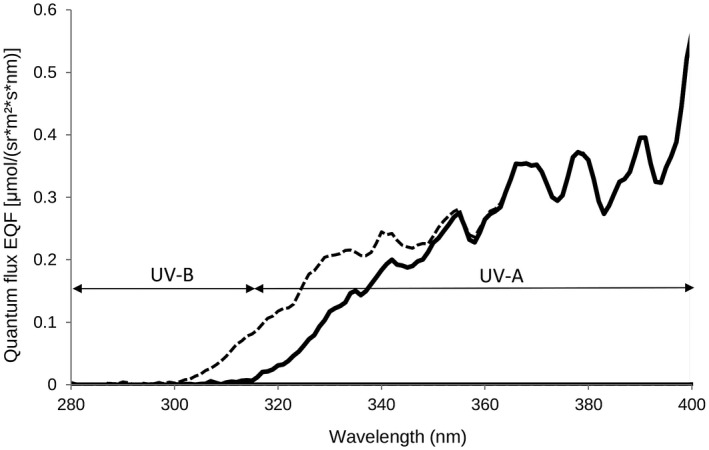
Ultraviolet (UV) light after entering the greenhouse (solid line) vs. UV light in the field (dashed line). E_QF_ = quantum flux.

**Figure 4:**
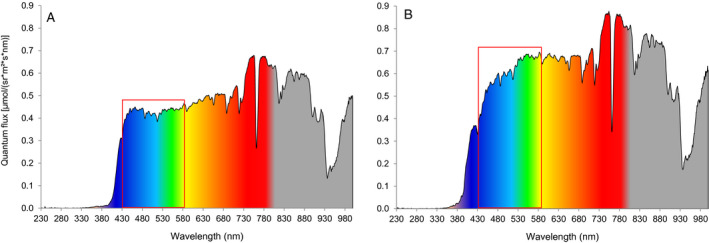
Light reflection [quantum flux (E_QF_) = µmol photons per steradian m^–2^ s^–1^ nm^–1^) in a greenhouse on (A) white ruffle device and (B) a white sticky plate that does not reflect ultraviolet A (UV‐A). The red square areas indicate the high blue peak in (A) compared to other colours due to UV‐A absorbance and re‐immittance to blue light by fluorescence vs. complete UV‐A absorbance without fluorescence in (B) leading to equivalent brightness of all colours.

In addition, both in the greenhouse and in the field, the wavelength spectrum of sunlight was measured (230–1 000 nm) with a broadband spectroradiometer Specbos 1211UV (Jeti Technische Instrumente, Jena, Germany). This was done to determine whether the greenhouse blocks certain wavelengths, which would possibly influence our results (Figure 3). Furthermore, the wavelength spectrum as reflected by the white ruffle material in the greenhouse was measured (Figure 4A) and, for comparison, that of a white trap (Figure 4B) in the greenhouse. The white trap is of unknown brand (supplied by Koppert Biological Systems) and has been tested previously in greenhouse and field tests for catching onion thrips. For measurements of sunlight reflection in the greenhouse compared to the field (Figure 3), a Spectralon disk, a certified reflectance standard (Labsphere, North Sutton, NH, USA), was used as a reference. Spectralon reflects 99.9% of all colours and is, as such, a reference for absolute light.

### Volatile compound

Methyl isonicotinate (purity 93%, batch 07906PZ; Sigma‐Aldrich, Castle Hill, Australia), was applied as a pure liquid on cotton dental rolls (38 mm long, 10 mm wide) at 1 ml per roll, approximately the maximum that can be applied without runoff. Rolls were placed directly above the devices. For the modified Lynfield device, the roll was placed on top of the lid of the container (Figure 1C), and for the ruffle devices, it was placed on the top end of the ruffle (Figure 1A). Nielsen et al. (2019) showed that the release rate from these cotton rolls is constant over time when compounds are applied in different amounts (0.5–2.5 ml). Thus, the amount applied is relevant for the duration of evaporation, but not for the release rate. The applied quantities evaporate in approximately 2 days. Lower and higher amounts of MI within a dispenser were shown to result in similar rates of thrips attraction, and no repellence (Davidson et al., 2008). Release rates of 1 ml MI at wind speeds of 0.6–1.2 m s^−1^ in a laboratory setting resulted in an actual MI concentration of 14.9 ng l^−1^ of air (van Tol et al., 2012).

### Thrips counts and identification

The total number of adult thrips caught on each device was established after the end of each sampling period (see below). Thrips were removed from the sticky devices using De‐Solv‐it (RCR International). All thrips were counted, and from each device, a maximum of 50 randomly selected thrips were mounted onto microscope slides and identified to species and sex. If the total number of thrips on a device was lower than 50, all were identified. In actual fact, all thrips per device were identified, with the exception of a few white ruffle traps in the field that contained 90–100 *T. tabaci*. All thrips were identified under 100× magnification according to Mound & Walker (1982) with allowances made for species that have entered New Zealand since that time.

### Field experiment

An experiment comparing thrips captures among the four trap types (three traps with/without MI and one control trap without MI) was performed on a grass field in Lincoln, New Zealand, at the New Zealand Institute for Plant and Food Research (43°38'16.2"S, 172°28'31.2"E). Two replicates (runs) were performed (on 11–13 and 21–25 March 2014). Temperature was 16–25 °C, and on 11–13 March wind speed was 7–32 km h^−1^ (prevailing direction East to Northeast, partly sunny to cloudy), whereas on 21–25 March wind speed was 9–33 km h^−1^ (prevailing direction East North East, scattered clouds) (https://www.timeanddate.com/weather/new‐zealand/christchurch/historic?month=3&year=2014). The second run was nearly 2 days longer than the first because thrips catches were low due to unfavourable weather conditions (low temperature and high wind speed) in the first few days. An extra 1 ml of MI was added to each dental roll after 48 h from the start of the second run.

**Figure 5:**
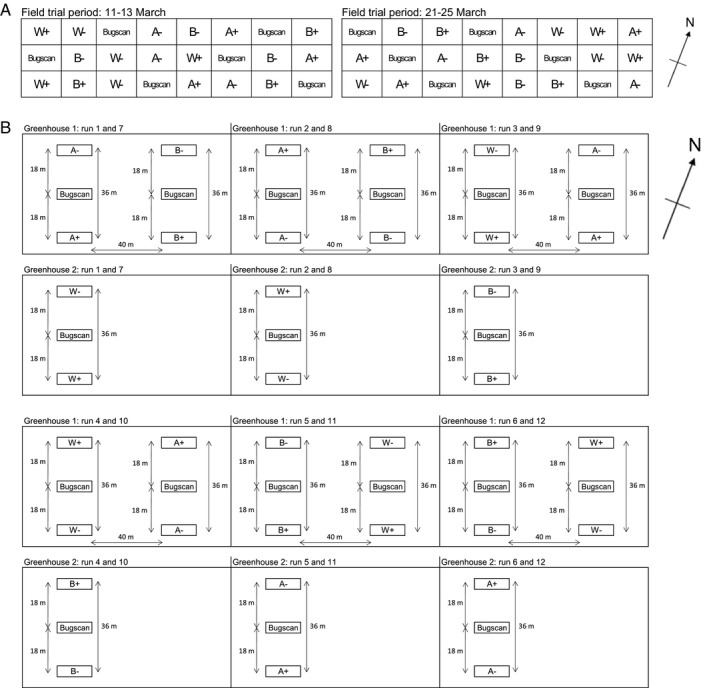
Layout of the (A) field and (B) greenhouse experiments. ‘+’ indicates the presence of methyl isonicotinate (MI), ‘−ʼ indicates the absence of MI. W, white ruffle device; B, blue ruffle device; Bugscan, control blue Bug‐scan sticky plate trap; A, blue modified Lynfield trap.

For each run, three replicates of each treatment (device type with or without MI) and six replicates of the control (Bug‐scan without MI) were laid out in a randomized block design, for a total of six and 12 replicates respectively (Figure 5A). Extra replicates of the Bug‐scan control traps without MI were included to more easily allow adjustment for possible spatial variation in thrips numbers, as the total test field was relatively large (experimental plot size: 280 m long × 80 m wide, within a field of 300 m long × 100 m wide) (Figure 5A). Devices were positioned 40 m apart, to minimize contamination by MI odour plumes (Teulon et al., 2014).

### Greenhouse experiment

An experiment comparing thrips captures among the four trap types (three trap types with/without MI and one control trap without MI) was performed in a rose (*Rosa* spp.) greenhouse in Halswell, New Zealand, at Moffatt’s Flower Company (43°35'42.4"S, 172°33'41.9"E). From 26 February–17 April 2014, 12 replicates (runs) were performed (two runs per week). Temperature in the greenhouse ranged from 16 °C during the night to 27 °C during the day. Wind speed varied from 2.2–2.9 km h^−1^. One control Bug‐scan trap (without MI) was placed in the middle between two other devices of the same type – one with and one without MI – in each row (sets) (Figure 5B). In this way, we compensated for possible variation in time and place of thrips density. Due to the size of the greenhouse compartments and the inability to position devices with and without MI sufficiently far apart to avoid possible cross‐contamination, we used a rotation schedule of treatments in time (runs) to compensate partially for the effect of location.

Runs were performed in two greenhouses. Greenhouses 1 and 2 (60 m long, 56 m wide) were located next to each other and separated by a plastic wall and a door. Each run comprised one replicate of each of the six treatments (white ruffle, blue ruffle, and modified Lynfield trap, all with/without MI), and three replicates of the control traps (without MI). Two sets were located in greenhouse 1 and one set in greenhouse 2, using the maximum width of the crop in the glasshouse (56 m), with a 10 m buffer zone on both ends to prevent edge effects. For each set, the devices were 18 m apart, thus the distance between each device without vs. with MI was 36 m. Distance between sets was 40 m. Any effect of MI on the control traps was assumed to be consistent between the runs, as control traps were always located between the pair of treatment traps. After each run, the position of the devices with and without MI in each set was swapped. Within a run, all devices without MI were located in the same position within all three sections (e.g., all at the rear of the greenhouse), and thus so were all devices with MI (e.g., all at the front of the greenhouse). This was done to minimize possible cross‐contamination of devices with and without MI. In each successive pair of runs, positions were rotated. New traps were used for each run.

Eight runs lasted 48 h and four runs lasted 65 h (run 1: 26–28 February; run 2: 3–5 March; run 3: 7–10 March; run 4: 11–13 March; run 5: 17–19 March; run 6: 21–24 March; run 7: 26–28 March; run 8: 31 March–2 April, run 9: 4–7 April; run 10: 8–10 April; run 11: 11–14 April; and run 12: 15–17 April). The longer runs were a result of inaccessibility to the greenhouses over weekends. After each run, there was a minimum break of 1 day, to allow any volatile MI in the greenhouse to dissipate.

### Statistical analysis

All analyses were performed with GenStat (GenStat Committee, 2013). Data (total thrips numbers) for both runs of the field experiment were analysed together, as were data for all 12 runs of the greenhouse experiment. To adjust for the varying surface areas of the devices, the thrips counts were scaled by the ratio of a trap’s area to the equivalent of 100 cm^2^ of the control Bug‐scan cylinder trap (Lynfield and ruffle device had 1.65 and 1.83× more surface area, respectively, than the Bug‐scan cylinder trap). This was done by including log[trap area/(100 × control trap area)] as an offset (McCullagh & Nelder, 1989). This is a parameter‐less explanatory variable, which allows the analysis of the measured counts, but assesses differences in the counts relative to device area, along with estimates for species/sexes derived from subsamples. For the ruffle devices, we calculated the surface of the cylindrical form of the ruffle for the comparison between devices. The data were analysed with a hierarchical generalized linear model approach (Lee et al., 2006), which is a mixed‐model extension for generalized linear models (McCullagh & Nelder, 1989). Fixed effects (treatments, contrasts between device types, colours, MI, and their interactions) were included with a Poisson distribution with a logarithmic link, and random effects (run, block, and test section) with a gamma distribution, also with a logarithmic link. The importance of random effects was assessed with a χ^2^ test of the change in deviance on dropping the effect, as implemented with the HGRTEST procedure. Fixed effects were assessed similarly, using Proc HGFTEST. The need for a separate estimate of dispersion for the two field trials or the 12 greenhouse runs was also assessed using a change in deviance test. In the final models, only important random effects were included. Separate dispersions were found to be required for all sets of data analysed, so they were included in the final models.

Trap areas were calculated as follows.

Blue and white ruffle devices: area of cylinder surface + area of top and bottom of cylinder = (2πr) × h + 2 × (πr^2^), where r = radius (2 cm) and h = height (30 cm). Thus, area = 401.92 cm^2^.

Lynfield trap: area of cylindrical beaker + bottom of beaker + lid of beaker = (2πr_1_) × h + (πr_2_^2^) + (πr_3_^2^), where r_1_ = 3.5 cm, r_2_ = 4.25 cm, r_3_ = 5.25 cm, and h = 10 cm. Area = 363.06 cm^2^.

Control trap: area of sticky plate curved into a cylinder = (2πr) × h, where r = 3.5 cm and h = 10 cm. Area = 219.80 cm^2^.

Species/sexes included in the analysis were those present in sufficiently large numbers to allow meaningful analysis, with a mean of >2 per trap. For the field data, these were *T. tabaci* females and *Thrips obscuratus* (Crawford) females, and for the greenhouse trials, *F. occidentalis* males and females. These data were analysed similar to the total thrips numbers, except that the offset to adjust for device area was modified by adding log (number identified/total thrips number), thereby adjusting both for differing device areas and sub‐sampling for identification.

In the results, predicted means for each device type are given, along with 95% confidence limits. These were obtained on the link (log) scale, using an offset of 0, and then converted to the original count scale for presentation.

## Results

### Spectral reflectance of trap material

The white ruffle device had a peak reflection in the ultraviolet A (UV‐A) around 365 nm, and the blue devices had very little UV‐A reflection (Figure 2). The UV‐A peak is caused by optical brighteners in the white fabric, which transmit the high‐energy UV‐A light to the lower‐energy blue light. This is shown by comparing the reflectance of the fluorescing white ruffle with the non‐fluorescing white plate (Figure 4A vs. 4B); more blue light was reflected on the white ruffle than on the white plate. The blue Bug‐scan and modified Lynfield devices had a near identical pattern, with a peak in the blue and a relatively high peak in the green/yellow range. The blue ruffle had a relatively low brightness and a peak in the blue/green range. Light reflection measurements indicated that 90% of the insect‐visible UV‐A (315–400 nm) was transmitted in the greenhouse compared to 100% in the field, i.e., the greenhouse glass blocked 10% of the UV‐A (Figure 3). Other colours all entered the greenhouse through the glass roof in the same proportions as in the field (data not shown). The greenhouse glass blocked 90% of the UV‐B (280–315 nm) (Figure 3).

### Field experiment

In total, 1 760 thrips were caught, of which 913 in the first and 847 in the second run. Over both runs, most thrips identified were *T. tabaci* females (76%). *Thrips obscuratus* females (9%) and males (3%) accounted for a small percentage of captures. Approximately 5% of captures consisted of *Anaphothrips obscurus* (Müller) females, and 4 and 2% were *Tenothrips frici* (Uzel) females and males respectively. Results are presented for *T. tabaci* and *T. obscuratus* females only (Table 1). In Table 2 the percentage of *T. tabaci* as a percentage of the mean total thrips per device is shown to indicate the different catch of this thrips species in response to the presence or absence of MI to the various devices.

**Table 1:** Mean number (+ 95% confidence interval) of female *Thrips tabaci* and *T. obscuratus* caught on four trap types in the absence (−) or presence (+) of methyl isonicotinate (MI) in the field experiment. Values represent mean count equivalent to an area of 100 cm^2^ of the control Bug‐scan sticky plate trap. Data of two runs of the experiment are pooled

Trap type	*T. tabaci*		*T. obscuratus*	
	−MI	+MI	−MI	+MI
Control	3.62 (2.52–5.21)		0.21 (0.08–0.54)	
Lynfield	0.44 (0.10–1.85)	7.46 (5.26–10.58)	0.00	0.41 (0.16–1.04)
White ruffle	9.51 (7.11–12.72)	23.49 (17.78–31.02)	0.30 (0.11–0.85)	5.19 (3.58–7.53)
Blue ruffle	0.88 (0.34–2.25)	6.10 (4.31–8.63)	0.00	0.59 (0.29–1.19)

**Table 2:** Mean percentage of *Thrips tabaci* females caught on four trap types in the field experiment in the absence (−) or presence (+) of methyl isonicotinate (MI). Values represent a percentage of the mean total thrips caught per device

Trap type	−MI	+MI
Control	86.3	
Lynfield	47.9	96.2
White ruffle	92.0	92.0
Blue ruffle	69.2	92.4

Overall, *T. tabaci* captures varied between the device types (χ^2^ = 59.7, d.f. = 2, P<0.001), with catches (numbers per unit area) on average highest for the white ruffle devices. More *T. tabaci* were caught in the presence of MI (χ^2^ = 40.6, d.f. = 1, P<0.001), but this effect varied with type and colour of the device (device type.ruffle colour.MI interaction: χ^2^ = 5.6, d.f. = 1, P = 0.021) (Table 1). White ruffle devices caught more thrips (3.9× with MI to 10.8× without MI) than blue ruffle devices (χ^2^ = 56.4, d.f. = 1, P<0.001), regardless of the presence of MI. Similar numbers of *T. tabaci* were caught on blue ruffle and blue modified Lynfield devices in the presence of MI, between 2–2.5× as many as were caught on the blue Bug‐scan control traps (without MI), and 7–17× more than were caught on the blue ruffle and modified Lynfield devices without MI (Table 1).

Few *T. obscuratus* were found on the devices in the absence of MI, with an estimated mean number of 0–0.30 per 100 cm^2^ area unit. There was little effect of MI on the trap captures of the blue ruffle and modified Lynfield devices, but a strong effect of MI on the capture of white ruffle devices (χ^2^ = 21.8, d.f. = 1, P<0.001) – the white ruffle trap with MI captured more than 10× more *T. obscuratus* than the other devices (Table 1).

### Greenhouse experiment

In total, 2 416 thrips were caught, of which 1 711 were identified (71%); they belonged to two species: *F. occidentalis* (females and males) and *T. tabaci* (females only). Over the 12 trials, an estimated 42% were *F. occidentalis* males, 49% were *F. occidentalis* females, and 7.3% were *T. tabaci* females. The numbers of *T. tabaci* caught were relatively low, so data for this species were not analysed.

*Frankliniella occidentalis* counts (numbers per unit area) varied substantially with device type (males: χ^2^ = 113.5; females: χ^2^ = 114.8; both d.f. = 6, P<0.001), and counts for the white ruffle devices were much higher than for any of the other devices. White ruffle devices with MI caught a similar number of thrips as white ruffle devices without MI (Table 3).

**Table 3:** Mean number (+ 95% confidence interval) of male and female *Frankliniella occidentalis* caught on four trap types in the absence (−) or presence (+) of methyl isonicotinate (MI) in the greenhouse experiment. Values represent mean count equivalent to an area of 100 cm^2^ of the control Bug‐scan sticky plate trap. Data of 12 runs of the experiment are pooled

Trap type	Males		Females	
	−MI	+MI	−MI	+MI
Control	1.39 (0.67–2.86)		2.27 (1.39–3.72)	
Lynfield	0.43 (0.20–0.95)	0.91 (0.46–1.77)	0.40 (0.23–0.68)	0.55 (0.32–0.94)
White ruffle	6.68 (3.68–12.14)	7.12 (3.92–12.95)	5.18 (3.21–8.35)	5.98 (3.70–9.64)
Blue ruffle	0.58 (0.29–1.18)	1.61 (0.86–3.03)	0.71 (0.41–1.22)	1.62 (0.96–2.73)

On average, more *F. occidentalis* females as well as males were caught on devices with MI than without MI (males: χ^2^ = 9.9, P = 0.002; females: χ^2^ = 3.4, P = 0.066; both d.f. = 1). However, this effect varied with type of device for both sexes (device type*MI interaction: (males: χ^2^ = 9.7, P = 0.008; females: χ^2^ = 5.5, P = 0.064; both d.f. = 2) – for males the MI effect was relatively strong with the blue ruffle (+MI/−MI = 2.8×) and the modified Lynfield (2.1×) devices, whereas for females it was strong with the blue ruffle device (2.3×) only. On average, white ruffle devices caught more thrips (both sexes) than blue ruffle or modified Lynfield devices, and than the control traps (males: χ^2^ = 93.4; females χ^2^ = 100.5; both d.f. = 2, P<0.001). The blue ruffle and modified Lynfield devices baited with MI caught similar numbers of male *F. occidentalis* as the control trap without MI, and fewer females that the control (Table 3).

## Discussion

The concept of auto‐dissemination relies on providing stimuli that elicit behavioural responses of pest insects that include attraction, landing on a device, and encouragement to walk around to pick up EPF spores, before leaving to disperse the spores to conspecifics, and eventually succumbing to infection from the EPF (Cork, 2016). In this study, we investigated the efficacy of three devices to improve insect attraction, i.e., the first part of auto‐dissemination for thrips management, in both greenhouse and field experiments. These traps introduced a range of new characteristics (enhanced colour, increased surface area) and interactions with a semiochemical (MI) that may be useful for improving outcomes for lure‐and‐infect for thrips, a management strategy that has not been fully exploited for these insects. We chose white and blue and not yellow as colours in our experiments, as we were interested in comparing both *T. tabaci* and *F. occidentalis* to these colours – i.e., *T. tabaci* is predominantly attracted to white and blue but variable to yellow, and *F. occidentalis* predominantly to blue and yellow and less to white (Brødsgaard, 1989; Natwick et al., 2007). Both species occur widespread in New Zealand (Mound & Walker, 1982). Unfortunately, in the greenhouse, we caught mainly *F. occidentalis* and the captures of *T. tabaci* were too low for a useful comparison between species. In the field, no *F. occidentalis* were caught.

Three naturally occurring thrips species landed in larger numbers per area unit on a white ruffle device than on a blue ruffle or a modified Lynfield device. This was shown for *T. tabaci* and *T. obscuratus* in the field, and for *F. occidentalis* in the greenhouse. We found that the light spectrum was not different for greenhouse and field in the visual range of the western flower thrips eye (Matteson et al., 1992; Otani et al., 2014) or the onion thrips eye (Makabe et al., 2014) (both: 355–650 nm; note that in Figure 3 data are only shown up to 400 nm), and as such was not likely to have influenced the responses of these species. Only at values below 350 nm is UV increasingly blocked by greenhouse glass. Based on the proportion of *T. tabaci* and *F. occidentalis* captured on differently coloured traps, Röth et al. (2016) suggested that these two species might have different photoreceptor systems.

The white ruffle device is showing fluorescence of the white fabric, which had a higher than 100% reflection in the UV range. In practice, this leads to transmission of UV to blue. The white ruffle device has higher reflectance values in the 400–480 nm range than a not fluorescing white surface. Added optical brighteners (to make the white ruffle appear brighter) absorb energy from the electromagnetic spectrum in the non‐visible UV area and emit it in a wider spectrum than was absorbed (in the range 400–480 nm). Fluorescent colours may lead to increased thrips attraction (Jenser et al., 2010; Röth et al., 2016). In unpublished field tests, the white ruffle fabric placed directly onto a white plate caught similar numbers of thrips as the white plate alone, whereas the white ruffle alone caught substantially more thrips, suggesting that fluorescence was not the only factor contributing to the response of thrips to this fabric (RWHM van Tol, unpubl.). This illustrates that we still do not understand all factors involved in the response of thrips to coloured traps.

The blue Bug‐scan control trap caught more thrips per unit area than the blue ruffle trap. This may have been due to a difference in spectral reflectance. Compared to the blue ruffle trap, the Bug‐scan trap is brighter, and also reflects more light in the yellow/green range. Brightness and colour preference of thrips may differ by season (Yudin et al., 1987; Hoddle et al., 2002; Yang et al., 2015; Röth et al., 2016). Blue is most attractive at low intensity, whereas yellow becomes more attractive in the high‐intensity range. Blackmer et al. (2008) found that white and blue were more attractive than yellow, whereas Yudin et al. (1987) found white to be more attractive than blue or yellow. Few publications report a strong attraction to white in western flower thrips (equal as or stronger than blue) (Yudin et al., 1987; Gillespie & Vernon, 1990; Hoddle et al., 2002; Blackmer et al., 2008), and few report no attraction at all (Chen et al., 2004; Davidson et al., 2012). In a wind tunnel experiment that tested preference of a New Zealand strain of western flower thrips to various colours, more females were caught on a yellow sticky plate (55.2%) than on a blue (20.8%) or white (4.7%) plate (Davidson et al., 2012). In our experiments in New Zealand we found the white ruffles to be significantly more attractive than the blue ones. Apparently, other unknown factors play a role.

Thrips attraction to UV reflectance of white may be dependent on the species. *Thrips tabaci* were more attracted to non‐UV‐reflecting white material than to UV‐reflecting white or blue (Kirk, 1984), whereas in other studies, *F. occidentalis* were attracted to blue (Roditakis et al., 2001; Chen et al., 2004; Broughton & Harrison, 2012), non‐UV‐reflecting white (Hoddle et al, 2002), and UV‐reflecting white material (Matteson et al., 1992; Makabe et al., 2014). The effect of UV reflectance on thrips attraction to – or avoidance of – white remains unclear.

The increased surface area of the folded fabric ruffle device may eventually play an important role in developing an effective lure‐and‐infect device for thrips. A higher number (per unit area) of thrips landed on the ruffled fabric compared to other devices. For these comparisons, we calculated the surface of the cylindrical form, but the actual surface area of the trap is larger, which may lead to even more thrips encountering and taking up spores of an EPF. The ruffle fabric and its folds may also contribute to better contact with the spores because of the cryptic behaviour of western flower thrips, which may trigger thrips to move further into the folds. This warrants further investigation, as it is unclear which trap characteristics are responsible for the increased thrips attraction. Cage tests in the presence of EPF, conducted prior to this study, indicated that blue ruffles led to higher thrips infection rates with EPF than the modified Lynfield devices (GJ Messelink & RWHM van Tol, unpubl.). This increased infectivity of the ruffle device may be related to cryptic thrips behaviour and folded fabric, although it still needs further study. The much more attractive white ruffle may increase infection rate of thrips over that already observed for the blue ruffle.

In the present study, we observed that *T. tabaci* and *T. obscuratus* in the field displayed a stronger response to the thrips lure MI (higher numbers in traps with MI than without MI) than *F. occidentalis* in the greenhouse. However, it was not possible to determine whether this contradictory response occurred because of species‐specific behavioural responses or attributes of the environment, as no thrips species was found in sufficient numbers for comparisons between greenhouse and field trials. The effects of MI were variable. Addition of MI to the white ruffles in the greenhouse experiment did not increase trap captures of either male or female *F. occidentalis*; the visual response to the white ruffle alone appeared to account for the strong attraction. In contrast, in the field trials, *T. obscuratus* exhibited a strong response to MI when combined with the white ruffle, whereas attraction to the white ruffle without MI was not much higher than attraction to the control Bug‐scan trap. The white ruffle without MI caught more *T. tabaci* than the control Bug‐scan trap, and addition of MI increased *T. tabaci* capture even more. This strong interaction between the white ruffle device and MI for *T. obscuratus* suggests an odour‐induced visual response, as the white ruffle without MI was hardly attractive. The interaction between colour and odour appears to be not straightforward and may be species specific.

Visual and olfactory stimuli play a major role in eliciting behavioural responses from insects, including thrips (Terry, 1997; Blackmer & Cañas, 2005; Campbell & Borden, 2006; Davidson et al., 2012; Döring, 2014; Barragán‐Fonseca et al., 2019). In their search for food many insect species integrate visual information with olfactory information specific for the host plant (Cardé & Hagaman, 1984; Charlton & Cardé, 1990; Hollister et al., 1995; Mathieu et al., 1997; Teulon et al., 1999; Henneman et al., 2002; Raguso & Willis, 2005; Campbell & Borden, 2006; Muvea et al., 2014). It is mostly unclear whether and how chemical and visual stimuli are interacting in guiding insects to their target. Our results indicate that in the presence of MI, the vast majority of the captured thrips was *T. tabaci* in all trap types, whereas in the absence of the lure, *T. tabaci* made up the majority of captures only in white ruffle traps. This supports the importance of the visual aspect of the white ruffle for attraction of *T. tabaci* in the absence of MI, and indicates a difference in species distribution between trap type/lure combinations.

The design of an effective auto‐dissemination device for thrips will require stimuli that not only elicit an alighting response, but also encourage the insects to maximize their exposure to the EPF spores, before they leave the device and succumb to infection from the EPF. Preferably, after initial contact with the spores, the thrips are covered by spores, disperse, and infect conspecifics in the surrounding vegetation, before dying (i.e., auto‐dissemination). For each thrips species, more detailed research is needed to understand the factors that could result in such a sequence of events. This study shows that we can improve the device design, using visual and olfactory cues, to encourage landing of thrips, which is necessary for uptake of EPF spores. In follow‐up research, we will investigate trap leaving behaviour. Furthermore, clarifying the complex responses of different thrips species to different colours and shapes, as well as the roles of UV‐A and chemical lures in attracting thrips, is essential not only to improve traps for lure‐and‐infect, but also for monitoring and mass‐trapping of thrips.
